# Interpectoral and Pectoserratus Plane Block vs. Local Anesthetic Infiltration for Partial Mastectomy: A Prospective Randomized Trial

**DOI:** 10.1155/2024/9989997

**Published:** 2024-03-20

**Authors:** Patryk Eisler, Stephan Zimmermann, Ragnar Henningsson

**Affiliations:** ^1^Department of Anesthesia and Intensive Care, Central Hospital Karlstad, Karlstad, Sweden; ^2^Department of Anesthesia, Spital Grabs, Grabs, Switzerland

## Abstract

**Background:**

Patients undergoing breast surgery are at risk of severe postoperative pain. Several opioid-sparing strategies exist to alleviate this condition. Regional anesthesia has long been a part of perioperative pain management for these patients.

**Aim:**

This randomized study examined the benefits of interpectoral and pectoserratus plane block (IPP/PSP), also known as pectoralis nerve plain block, compared with advanced local anesthetic infiltration.

**Methods:**

We analyzed 57 patients undergoing partial mastectomy with sentinel node dissection. They received either an ultrasound-guided IPP/PSP block performed preoperatively by an anesthetist or local anesthetic infiltration performed by the surgeon before and during the surgery.

**Results:**

Pain measured with the numerical rating scale (NRS) indicated no statistically significant difference between the groups (IPP/PSP 1.67 vs. infiltration 1.97; *p* value 0.578). Intraoperative use of fentanyl was significantly lower in the IPP/PSP group (0.18 mg vs 0.21 mg; *p* value 0.041). There was no statistically significant difference in the length of stay in the PACU (166 min vs 175 min; *p* value 0.51). There were no differences in reported postoperative nausea and vomiting (PONV) between the groups. The difference in postoperative use of oxycodone in the PACU (*p* value 0.7) and the use of oxycodone within 24 hours postoperatively (*p* value 0.87) was not statistically significant.

**Conclusions:**

Our study showed decreased intraoperative opioid use in the IPP/PSP group and no difference in postoperative pain scores up to 24 hours. Both groups reported low postoperative pain scores. This trial is registered with NCT04824599.

## 1. Introduction

Breast cancer with concomitant surgery carries a significant risk of psychological as well as physical complications [1, 2]. Developments in both surgical technique and anesthesia and analgesia have improved pain control and decreased the incidence of postoperative nausea and vomiting (PONV). Regional anesthesia has become a cornerstone of perioperative pain management during breast cancer surgery, resulting in a reduced need for opioids [3, 4]. The Enhanced Recovery After Surgery (ERAS®) society recommends multimodal opioid-sparing analgesia, including regional analgesia techniques to minimize opioid side effects such as delayed mobilization [5]. It is even postulated that the use of local anesthetics together with opioid-sparing analgesia can improve prognosis in cancer patients by reducing the risk of metastases [6–9]. Opioids have been shown to negatively influence natural killer (NK) cells responsible for human cell defense against tumor cell spreading during surgery.

Paravertebral anesthesia is considered the gold standard for optimal pain control in breast surgery [10]. The ability to administer repeated doses of local anesthetics through a paravertebral catheter allows pain treatment corresponding to patients' needs [11, 12]. However, the risk of complications such as pneumothorax or inadvertent epidural or intrathecal injection [13, 14] has led to the emergence of other ultrasound-guided techniques.

Pectoral nerves plane block (PECS), first described by Blanco [15, 16], is among more recent adjuncts in perioperative pain management in breast and thoracic surgery and in thoracic trauma [17, 18]. According to newly published recommendations for nomenclature, the PECS-block is referred to as interpectoral and pectoserratus plane block (IPP/PSP) [19].

Several studies have shown good results when IPP/PSP is compared with other analgesic strategies [20–23]. A recent review article published by Wong et al. underlines the important role of regional anesthesia in breast surgery [24]. However, in patients undergoing less traumatic breast surgery, in particular partial mastectomies (lumpectomies) without axillary lymph node dissection, the benefit of regional anesthesia as compared to local anesthetic (LA) infiltration is less clear.

In this randomized controlled study, we hypothesized that IPP/PSP blockade would be associated with a decrease in systemic opioid utilization compared with when local anesthetics are injected by surgeons in the operating field during partial mastectomies. Other outcomes were postoperative pain scores measured by the numerical rating scale (NRS-pain), 24 hours' postoperative oxycodone consumption, the occurrence of PONV, and length of stay in the postanesthesia care unit (LOS-PACU).

## 2. Methods

This single-center prospective randomized controlled trial was performed between February 2021 and May 2022 at the Department of Anesthesia and Intensive Care, Central Hospital in Karlstad, Sweden (ClinicalTrials.gov identifier NCT04824599). The study was approved by the Swedish Ethical Review Agency (approval no. 2019-04475; approval date September 25, 2019).

The delay between receipt of ethical approval and start of the study was caused by organizational changes in our department in response to the SARS-CoV-2 virus pandemic.

Patients scheduled for partial mastectomy (lumpectomy) with a sentinel node dissection were eligible for the study. Exclusion criteria were planned frozen section examination, axillary lymph node dissection, reoperation, age <18 years or intellectual disability, severe chronic pain, local anesthetic allergy, present drug addiction, and pregnancy. Informed written consent was obtained from all patients who participated in the study. Patients were randomly allocated to “IPP/PSP” or “Infiltration” group using a simple randomization method (following a computer-generated randomization list (Research Randomizer (Version 4.0), https://www.randomizer.org). Allocation concealment was achieved using opaque, sealed envelopes. Neither personnel nor patients were blinded after assignment to intervention.

Patients were randomized to either receive an ultrasound-guided IPP/PSP block performed by the anesthetist with a wound infiltration performed by the surgeon at the end of the surgery or LA infiltration by the surgeon. All anesthetists conducting IPP/PSP blocks went through a training program to guarantee high quality of regional anesthesia. The program required repeated supervised performance of IPP/PSP block.

PONV risk was assessed using the Apfel score [25] during the preoperative anesthesiologic consultation. All patients included in the study received oral premedication with acetaminophen 1 g, meclozine 25 mg, and etoricoxib 90 mg (standard in our department).

For the ultrasound technique, a portable device with a linear probe was used (BK medical® Flex Focus 500 Ultrasound Machine, high-frequency linear probe 8870). The IPP/PSP blockade was performed using an ultrasound in-plane technique injecting a total of 2 mg/kg of ropivacaine 3.75 mg/ml in two locations specified by IPP/PSP blockade. At the end of the surgery, the surgeon infiltrated the surgical wound with an additional 1 mg/kg of ropivacaine 2 mg/ml.

Members of the LA infiltration group received the first subcutaneous infiltration of local anesthetic prior to scrubbing and incision (ropivacaine 3.75 mg/ml 1 mg/kg) and the second deeper infiltration after the lump removal (ropivacaine 3.75 mg/ml 2 mg/kg). The abovementioned advanced local anesthetic infiltration technique was developed by all three surgeons in charge of performing breast surgery. For patients with obesity class 2, defined as the body mass index >35, an adjusted body weight was used to calculate the ropivacaine dose [26–28]. Patients were anesthetized with propofol 1% 2 mg/kg and a standard dose of fentanyl of 0.1 mg with an additional dose administered at the discretion of the person delivering the anesthesia. The airway was maintained with a supraglottic device or tracheal intubation when indicated. The anesthesia was maintained using propofol administered with a target control infusion syringe pump (Alaris™ PK) and fentanyl given intermittently. After surgery, all patients were transferred to the PACU, where standardized data were collected. Pain and nausea were registered hourly until discharge from PACU with the use of a 10-point numerical rating scale (NRS). Rescue analgesia was available when NRS values were ≥4. If they met the discharge criteria for the PACU, patients were free to go home or could choose to stay at a rooming facility on the hospital premises for the first postoperative night. Telephone follow-up was scheduled on the first postoperative day as well as 30 days postsurgery. A standardized questionnaire was used, with a focus on pain scoring.

### 2.1. Statistics

Initially, the number of patients necessary for inclusion in the study (80) was estimated through analysis of similar studies performed in the field with the same endpoint [29]. Due to the COVID pandemic and because two involved surgeons and one anesthesiologist moved to another hospital, the study had to be stopped before all 80 patients could be included. Studies with similar topics and design achieved a statistical power of 0.90, with (*n* = 30) [4, 30, 31] able to detect a 20% reduction in opioid consumption. This supports the decision to close the trial early, at the level achieved with 60 included patients.

For the statistical analysis, we used Student´s *t*-test for independent data (fentanyl dose, local anesthetic dose, NRS pain score). For nonparametric data, the Mann–Whitney *U* test was used (postoperative oxycodone consumption). The critical level of probability was *p* < 0.05. The tests used were two-tailed. Distribution spread is disclosed as the median interquartile range (IQR). Software used for the analysis was MS Office 2010 and R package for statistical computing (https://www.r-project.org/)

## 3. Results

**Figure 1:**
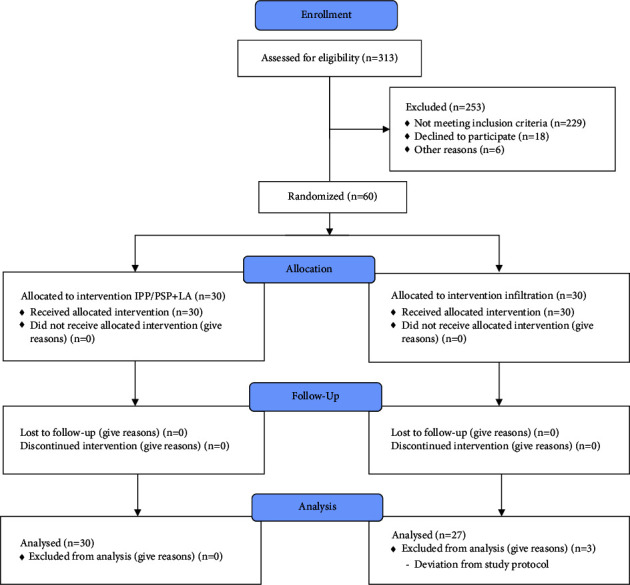
Consolidated standards for reporting of trials (CONSORT) diagram of the trial.

Out of 313 patients screened for eligibility, 229 did not meet the inclusion criteria, 18 declined to participate, and another 6 were excluded for various reasons, such as changes in the operation technique prior to randomization or lack of staff familiar with the study protocol (Figure 1). Sixty (*n* = 60) patients were randomized to one of the intervention arms during the study. Thirty patients received IPP/PSP block, and thirty patients underwent LA infiltration. Data from 57 patients were analyzed (3 patients were excluded from the analysis due to serious protocol deviation). The enrollment and follow-up period were between February 2021 and May 2022. No harm or inadvertent reaction was registered during the trial.

The baseline demographics of each group can be seen in Table 1. There were no statistical differences between the groups with respect to age, BMI, or ASA class.

**Figure 2:**
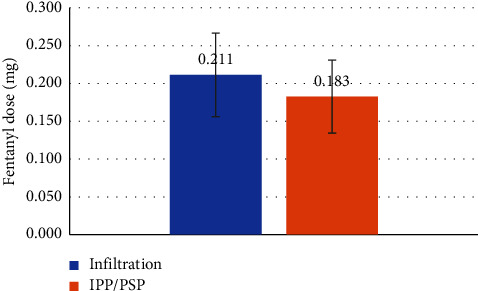
Comparison of intraoperative fentanyl doses between the groups during partial mastectomy (one received wound infiltration, and another group received interpectoral and pectoserratus plane block).

**Figure 3:**
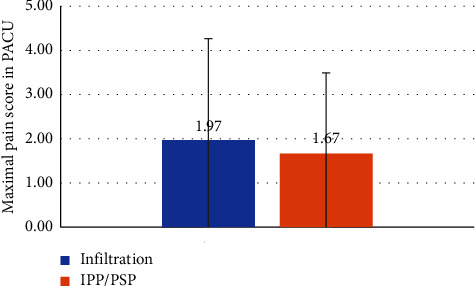
Comparison of the maximal pain score after partial mastectomy in the postanesthesia recovery unit between the two groups (one received wound infiltration, and another group received interpectoral and pectoserratus plane block).

**Figure 4:**
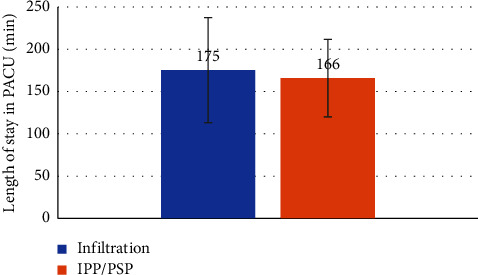
Comparison of length of stay in the postoperative care unit after partial mastectomy between the two groups (one received wound infiltration, and another group received intrapectoral and pectoserratus plane block).

Our findings demonstrate that although the fentanyl dose administered in the operating room was significantly lower in the IPP/PSP group (Figure 2) (0.18 mg vs 0.21 mg, *p*-value 0.04) (median (IQR) 0.19 (0.15–0.20) vs. 0.18 (0.15–0.19)), secondary endpoints could not support the superiority of the IPP/PSP block. We were unable to demonstrate with statistical significance a superiority of the ultrasound-guided IPP/PSP block compared to advanced local anesthetic infiltration with regard to postoperative pain control (Figure 3): maximal NRS pain score 1.67 vs. 1.97; *p* value 0.57, (median (IQR) 1 (0–4) vs. 1 (0–2.5)); length of stay in PACU (LOS-PACU) (Figure 4) 166 min vs. 175 min, *p* value 0.50 (median (IQR) 172 (132–214) vs. 166 (125–187)); maximal pain 24 hours postsurgery (NRS pain score 3.0 vs. 2.4, *p* value 0.25) (median (IQR) 3 (2–4)) vs. 2 (0.8–4)), or postoperative oxycodone consumption in the first 24 hours postsurgery (2.83 vs. 2.67 mg, *p* value 0.87) (median (IQR) 0 (0–5) vs. 0 (0–5)).

**Figure 5:**
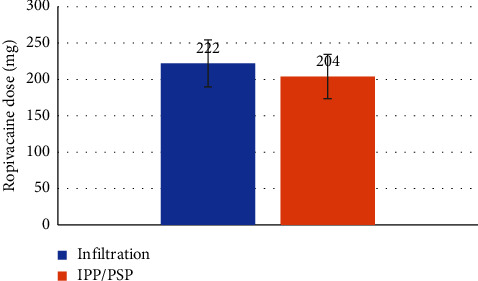
Comparison of ropivacaine doses between the two groups undergoing partial mastectomy (one received wound infiltration, and another group received interpectoral and pectoserratus plane block).

The ropivacaine dose was significantly lower in the IPP/PSP block group (Figure 5) 204 mg vs. 222 mg, *p* value 0.03) (median (IQR) 205 (178–227) vs. 218 (200–241)). The incidence of PONV was very low in both groups, with just three patients indicating nausea in the PACU. The follow-up after a month confirmed no significant pain in either group (NRS pain score 1.07 vs. 0.80, *p* value 0.48) (median (IQR) 0 (0–2) vs. 0 (0–2)).

## 4. Discussion

Our study showed no superiority of an ultrasound IPP/PSP block compared with local anesthetic injected perioperatively by surgeons trained in infiltration techniques in patients undergoing partial mastectomy in an ambulatory setting. Considering the results of our study, one could hypothesize that the local anesthetic injected by the surgeon was administered with more accuracy due to visual control. Anatomy of the IPP/PSP block spread is usually limited to lateral quadrants with medial parts of the breast not covered sufficiently by this block. However, as the total amount of local anesthetics was equal in both groups and the ultrasound-led IPP/PSP block is a standardized procedure, further research into the surgical infiltration in partial mastectomy is needed in order to fully explain the result.

Assuming there is no direct patient benefit for either of the methods, it can be postulated that advanced local infiltration has the benefit of reducing the resources needed in order to carry out the anesthesia, saving time and costs connected with the provision of IPP/PSP block. Another benefit of local anesthetic infiltration is the possible reduction of the existing but small risk of complications associated with pectoral plane blocks, such as pneumothorax [32].

Considering the scarcity of studies comparing IPP/PSP with local anesthesia for partial mastectomy in an ambulatory setting, our prospective randomized study attempts to address this gap in knowledge.

The significantly lower perioperative fentanyl dose in the IPP/PSP group could be explained with better perioperative analgesia due to the administered blockade and should be considered with the knowledge that the anesthesia personnel were unblinded regarding the allocation group of the patients. The benefit did not however translate to improvement in postoperative pain control. The mean value of maximal NRS pain in both groups was below 4 (and in fact, below 2), which generally is considered satisfactory with regard to pain control.

Although the length of stay in the PACU is an interesting endpoint considering the potential to improve the flow of patients in the PACU, we were unable to prove any statistically significant difference. LOS PACU is easily influenced by factors such as anesthesia technique, kind and technique of surgery, personnel experience, and organizational aspects, including the capacity to transfer patients to a ward. In our facility, most patients stay overnight at the hotel connected to the hospital.

Since the first publication by Blanco in 2011, several studies have examined the benefits of IPP/PSP [4, 21, 33]. Recently published PROSPECT guidelines [34] recommend administration of regional anesthesia for major breast surgery. Local anesthetic infiltration is recommended only as a supplement.

In the ambulatory setting, an effective postoperative pain treatment strategy is of highest priority. It has been documented in the ERAS society recommendations that opioid sparing is an important component of good and fast recovery after surgery [35]. Regional anesthesia has proven to be an effective alternative to opioids for postoperative pain [36]. A multimodal approach using acetaminophen, NSAID, betamethasone, and clonidine might reduce the importance of IPP/PSP compared to advanced local anesthetic infiltration.

The Society for Ambulatory Anesthesia (SAMBA) recently published a review regarding IPP/PSP in breast surgery [37]. They concluded that, although pectoral plane blocks seem to moderately reduce postoperative opioid use compared to systemic analgesia, there are no grounds to recommend pectoral plane blocks over local infiltration performed by the surgeon. This conclusion would support our findings.

### 4.1. Limitations

A main limitation of our study is the fact that the trial was stopped before the initially planned number of participants (*n* = 80) could be included. The rationale for concluding the study was presented in the statistics section. Another weakness of the study was the intervention's lack of blinding. This could have influenced the amount of opioids administered in the operating room. The analysis of intraoperative fentanyl use indicated a statistically significant difference, which should be considered with the knowledge that the anesthesia personnel were unblinded regarding the allocation group of the patients.

## 5. Conclusion

Our randomized study could not prove, with statistical significance, the superiority of IPP/PSP block over advanced wound infiltration performed by trained surgeons in patients undergoing lumpectomy. Both groups reported low pain scores.

## Figures and Tables

**Table 1:** Demographics.

	IPP/PSP + LA	Infiltration
Age (y)	61.2 ± 10.6	62.2 ± 10.1
Weight (kg)	70.8 ± 12	76.7 ± 17.3
BMI (kg/m^2^)	26.2 ± 4.1	28.0 ± 5.8
ASA class (*n*)		
ASA I	13	9
ASA II	15	16
ASA III	2	2

Mean values ± SD.

## Data Availability

The data that support the findings of this study are available from the corresponding author upon reasonable request.
